# Evaluation of the extracranial “multifocal arcuate sign,” a novel MRI finding for the diagnosis of giant cell arteritis, on STIR and contrast-enhanced T1-weighted images

**DOI:** 10.1186/s12880-024-01314-4

**Published:** 2024-06-05

**Authors:** Toshitada Hiraka, Yasuhiro Sugai, Yoshihiro Konno, Yuuki Toyoguchi, Yoshie Obata, Shin Ohara, Akiko Shibata, Yusuke Takeda, Koichi Nishitsuka, Kazunobu Ichikawa, Masafumi Watanabe, Yukihiko Sonoda, Masafumi Kanoto

**Affiliations:** 1 Department of Radiology, Division of Diagnostic Radiology, Yamagata University Faculty of Medicine, 2-2- 2 Iida-Nishi, Yamagata, 990-9585 Japan; 2 Department of Ophthalmology and Visual Sciences, Yamagata University Faculty of Medicine, 2-2-2 Iida- Nishi, Yamagata, 990-9585 Japan; 3 Department of Cardiology, Pulmonology, and Nephrology, Yamagata University Faculty of Medicine, 2-2-2 Iida-Nishi, Yamagata, 990-9585 Japan; 4 Department of Neurosurgery, Yamagata University Faculty of Medicine, 2-2-2 Iida-Nishi, Yamagata, 990- 9585 Japan; 5 Department of Diagnostic Radiology, Yamagata University Faculty of Medicine, 2-2-2 Iida-nishi, Yamagata, 990-9585 Japan

**Keywords:** Giant cell arteritis, MRI, STIR

## Abstract

**Background:**

While early diagnosis of giant cell arteritis (GCA) based on clinical criteria and contrast-enhanced MRI findings can lead to early treatment and prevention of blindness and cerebrovascular accidents, previously reported diagnostic methods which utilize contrast-enhanced whole head images are cumbersome. Diagnostic delay is common as patients may not be aware of initial symptoms and their significance. To improve current diagnostic capabilities, new MRI-based diagnostic criteria need to be established. This study aimed to evaluate the “multifocal arcuate sign” on short tau inversion recovery (STIR) and contrast-enhanced T1-weighted (CE-T1W) images as a novel extracranial finding for the diagnosis of GCA.

**Methods:**

A total of 17 consecutive patients (including five with GCA) who underwent CE-T1W and whole-brain axial STIR imaging simultaneously between June 2010 and April 2020 were enrolled. We retrospectively reviewed their MR images. The “multifocal arcuate sign” was defined as “multiple distant arcuate areas with high signal intensity in extracranial soft tissues such as subcutaneous fat, muscles, and tendons.” Extracranial abnormal high-signal-intensity areas were classified as “None,” when no lesions were detected; “Monofocal,” when lesions were detected only in one place; and “Multifocal,” when lesions were detected in multiple places. The sensitivity, specificity, positive predictive value (PPV), and negative predictive value (NPV) of “Multifocal” areas were calculated using cross tabulation. Fisher’s exact test was used to compare “Multifocal” areas in five patients with GCA and those with other diseases. In addition, mean Cohen’s kappa and Fleiss’ kappa statistics were used to compare inter-reader agreement.

**Results:**

The sensitivity, specificity, PPV, and NPV of the “multifocal arcuate sign” in patients with GCA were 60%, 92–100%, 75–100%, and 85–86%, respectively. Significantly more patients with GCA had “Multifocal” areas compared to those with other diseases (Fisher’s exact test, *p* = 0.008–0.027). Mean Cohen’s kappa and Fleiss’ kappa for inter-reader agreement with respect to the five GCA patients were 0.52 and 0.49, respectively, for both STIR and CE-T1W sequences.

**Conclusions:**

The new radiologic finding of “multifocal arcuate sign” on STIR and CE-T1W images may be used as a radiologic criterion for the diagnosis of GCA, which can make plain MRI a promising diagnostic modality.

## Introduction

Giant cell arteritis (GCA) is a granulomatous arteritis of large arteries and branches that come off the aorta, with the most common site being extracranial arteries, especially the superficial temporal artery, typically seen in older people [1]. The incidence of GCA is the highest in European populations (about 10–20 cases per 100,000 persons aged ≥ 50 years), and is markedly lower in American populations of Asian or African descent (about 1 case per 100,000 persons) [2]. Polymyalgia rheumatica (PMR) is a common and frequently overlapping condition of GCA [3].

Characteristic symptoms of GCA include temporal artery tenderness, and patients also develop nonspecific symptoms such as headache, fever, weight loss, and arthralgia. Arteritis causes luminal occlusion and therefore leads to ischemic complications, including arterial anterior ischemic optic neuropathy, which results in rapid and irreversible unilateral or bilateral blindness in roughly 10–20% of patients [1, 3]. GCA is also associated with complications such as aortic dissection, aneurysm formation [3], myocardial infarction, and cerebrovascular accidents [4]. Prevention of these serious complications requires early diagnosis and treatment initiation.

While the importance of early diagnosis and treatment of GCA had been established, a systematic review in 2017 concluded that early diagnosis of GCA is difficult [5]. One study reported that 15 of 17 patients with GCA who visited a headache clinic had not been diagnosed with GCA by their previous physicians [6]. Failure to promptly seek medical attention also results in diagnostic delay, as the significance of symptoms, such as jaw claudication and temporal artery abnormality, in GCA is often under-recognized by patients. Primary care physicians need to identify early symptoms of GCA, many of which are frequently non-specific despite the relative rarity of the disease. The high prevalence of similar symptoms in the general consulting population also contributes to the delay in diagnosing GCA [5, 6].

GCA has been diagnosed according to the classification criteria proposed by the American College of Rheumatology in 1990 [7]. Invasive histological findings of biopsy specimens from the superficial temporal artery are important for a GCA diagnosis, since clinical and laboratory findings are non-specific. Although imaging analysis is not a diagnostic criterion for GCA, several reports have described the usefulness of MRI (sensitivity: 78.4–80.6%, specificity: 90.4–97.0%) [8, 9], FDG-PET (sensitivity: 56–80%, specificity: 89–98%) [10], and ultrasonography (sensitivity: 40–87%, specificity: 79–100%) [10, 11] findings for diagnosing GCA. Contrast-enhanced MRI is important for confirming the presence of lesions before initiating steroid therapy, and is also useful in determining indications for tissue biopsy and differentiating between GCA and other diseases, such as anti-neutrophil cytoplasmic antibody (ANCA) associated vasculitis. In Japan, plain MRI is often performed as a screening test for non-specific headache when GCA, ANCA associated or other vasculitis, and meningitis are not suspected. The diagnostic ability of non-contrast-enhanced MRI for GCA, however, has never been examined. In previous studies, imaging findings of contrast-enhanced MRI that suggest GCA included the presence of a contrast effect around small arteries in the whole head [9]. However, detailed examination of whole head images is difficult, and previously reported diagnostic methods which utilize contrast-enhanced images is cumbersome. Therefore, to improve current diagnostic capabilities, new MRI findings need to be established that aid in the diagnosis of GCA along with the clinical criteria and existing MRI methods.

In some patients with GCA, we found multiple distant arcuate areas with high signal intensity on short tau inversion recovery (STIR) images and enhancement on contrast-enhanced images of extracranial soft tissues such as subcutaneous fat, muscles, and tendons. We hypothesized that changes in extracranial soft tissues, such as subcutaneous fat, muscles, and tendons, may occur in GCA. We identified an abnormal extracranial soft tissue intensity in brain MRI and refer to it herein as the “multifocal arcuate sign.” The present study aimed to evaluate the multifocal arcuate sign on both plain and contrast-enhanced images.

## Materials and methods

### Participants

This retrospective study was approved by the Ethical Review Committee of Yamagata University Faculty of Medicine (Registration Number: 2020 − 109). The need for Informed Consent was waived by the Ethical Review Committee of Yamagata University Faculty of Medicine because the study was pure observational. Informed consent was waived and the opt-out agreement was applied by the Ethical Review Committee of Yamagata University Faculty of Medicine.

Participants were 17 consecutive patients (mean age ± SD, 47.9 ± 25.6 [range: 0–87] years; 4 men and 13 women) who underwent contrast-enhanced MRI and whole-brain axial STIR imaging simultaneously at our hospital between June 2010 and April 2020. Very few patients underwent contrast-enhanced MRI and whole-brain axial STIR imaging simultaneously, with only 17 consecutive patients doing so over the course of 10 years. These 17 patients included five patients with GCA, one with aortitis syndrome and headache, three with epilepsy, three with leukemia (one with chloroma, one in remission), two with postoperative wound infection (meningioma), one with extracranial hemangioma, one with postoperative temporal hemangioma, and one with bone metastasis (gastric cancer) (Table 1). All five patients with GCA were diagnosed on the basis of clinical symptoms according to the classification criteria proposed by the American College of Rheumatology in 1990 and radiological examinations including MRI. One of the patients also underwent temporal artery biopsy, which revealed pathological inflammatory findings.

**Table 1:** Patient demographics and clinical characteristics

Characteristics	Value
Study period	June 2010 to April 2020
Number of patients	17
Sex (male: female)	4:13
Mean age ± SD (range)	47.9 ± 25.6 (0–87)
Clinical manifestations	
GCA	5
GCA with TAB	1
GCA without TAB	4
Aortitis syndrome with headache (not diagnosed as GCA)	1
Epilepsy	3
Leukemia	3
Postoperative wound infection (meningioma)	2
Extracranial hemangioma	1
Postoperative temporal hemangioma	1
Bone metastasis (gastric cancer)	1

*Note* GCA = giant cell arteritis, TAB = temporal artery biopsy

### MRI acquisition

All scans were performed using a 3.0 Tesla MR imaging unit (Achieva and Achieva dStream, Philips, the Netherlands) with a dS Head 32ch coil. The following parameters were used for each sequence: (1) STIR: repetition time (TR)/echo time (TE) 3400/40 ms; field of view (FOV) 240 × 240 mm; IR delay 210 ms; SENSE P-reduction 1.3; flip angle 90 degrees; slices 18; slice thickness 6.0 mm; scan time 4 min 12 s; (2) T1-turbo field echo [T1-TFE]: TR/TE 11/6.3 ms; FOV 240 × 240 mm; SENSE P-reduction 1.7, S-reduction 1.5; flip angle 8 degrees; slices 200; slice thickness 0.8 mm; scan time 6 min 21 s; and (3) 3D-T1-volume isotropic TSE [T1-VISTA]: TR/TE 400/19 ms; FOV 240 × 240 mm; SENSE P-reduction 2.0, S-reduction 2.0; flip angle 80 degrees; slices 290; slice thickness 1.2 mm (spacing between slices 0.6 mm); spectral presaturation with inversion recovery (SPIR) fat suppression; scan time 4 min 42 s. A gadolinium-based contrast medium (0.1 mmol/L) was used at a dose of 0.2 ml/kg. We had scanned fat-suppressed STIR images for detecting inflammatory findings due to GCA/PMR before administering a gadolinium-based contrast medium. (4) T2WI: TR/TE 3300/90 ms; FOV 240 × 240 mm; SENSE P-reduction 1.7; flip angle 90 degrees; slices 18; slice thickness 6.0 mm; scan time 2 min 19 s, (5) FLAIR: TR/TE 9000/90 ms; FOV 240 × 240 mm; IR delay 2500 ms; SENSE no; flip angle 90 degrees; slices 18; slice thickness 6.0 mm; scan time 2 min 42 s, (6) T1WI: TR/TE 350/10 ms; FOV 240 × 240 mm; SENSE no; flip angle 80 degrees; slices 18; slice thickness 6.0 mm; scan time 2 min 21 s, (7) DWI: TR/TE shortest/80 ms; FOV 240 × 240 mm; SENSE P-reduction 2.5; flip angle 90 degrees; slices 18; slice thickness 6.0 mm; spectral attenuated with inversion recovery (SPAIR) fat suppression; scan time 0 min 41 s, (8) T2*: TR/TE shortest/23 ms; FOV 240 × 240 mm; SENSE P-reduction 1.2; flip angle 18 degrees; slices 18; slice thickness 6.0 mm; scan time 1 min 58 s, and (9) MRA: TR/TE 23/3.5 ms; FOV 210 × 210 mm; SENSE P-reduction 2.4; flip angle 20 degrees; slices 120; slice thickness 0.6 mm; scan time 3 min 42 s.

All patients underwent conventional sequences including whole brain STIR and CE-T1W imaging (T1-TFE and/or T1-VISTA). The majority of contrast-enhanced sequences scanned was T1-TFE, but some cases of GCA had been scanned only by T1-VISTA. Among the five GCA patients, one (Case 8) underwent only T1-TFE, two (Cases 14 and 16) underwent only T1-VISTA, and two (Cases 10 and 11) underwent both T1-TFE and T1-VISTA. IR pulse was used for fat suppression on STIR. In T1-TFE, the fat signal is reduced by the IR pulse, which is used to improve white matter/gray matter contrast, but has a fat suppression effect. Unevenness due to suppression from the IR pulse is likely minimal. Moreover, fat suppression on T1-VISTA was achieved by SPIR, which is weak in uneven magnetic fields but can be used relatively stably for the head with minimal unevenness of suppression. There were no cases in which fat suppression was not homogeneous.

### Definition of the “multifocal arcuate sign”

We defined the extracranial “multifocal arcuate sign” as multiple distant arcuate areas with high signal intensity on STIR images and enhancement on contrast-enhanced images of extracranial soft tissues such as subcutaneous fat, muscles, and tendons. The high-signal-intensity enhancement area was observed mainly in fatty tissue around arteries and in soft tissue, such as the aponeurosis and temporal muscle, as linear or arcuate regions of uneven thickness. In the interpretation experiment, we classified patients with no arcuate sign as “None,” those with an arcuate sign in a single site as “Monofocal,” and those with arcuate signs in multiple distant lesions of extracranial soft tissues as “Multifocal.”

### Image evaluation

**Fig. 1:**
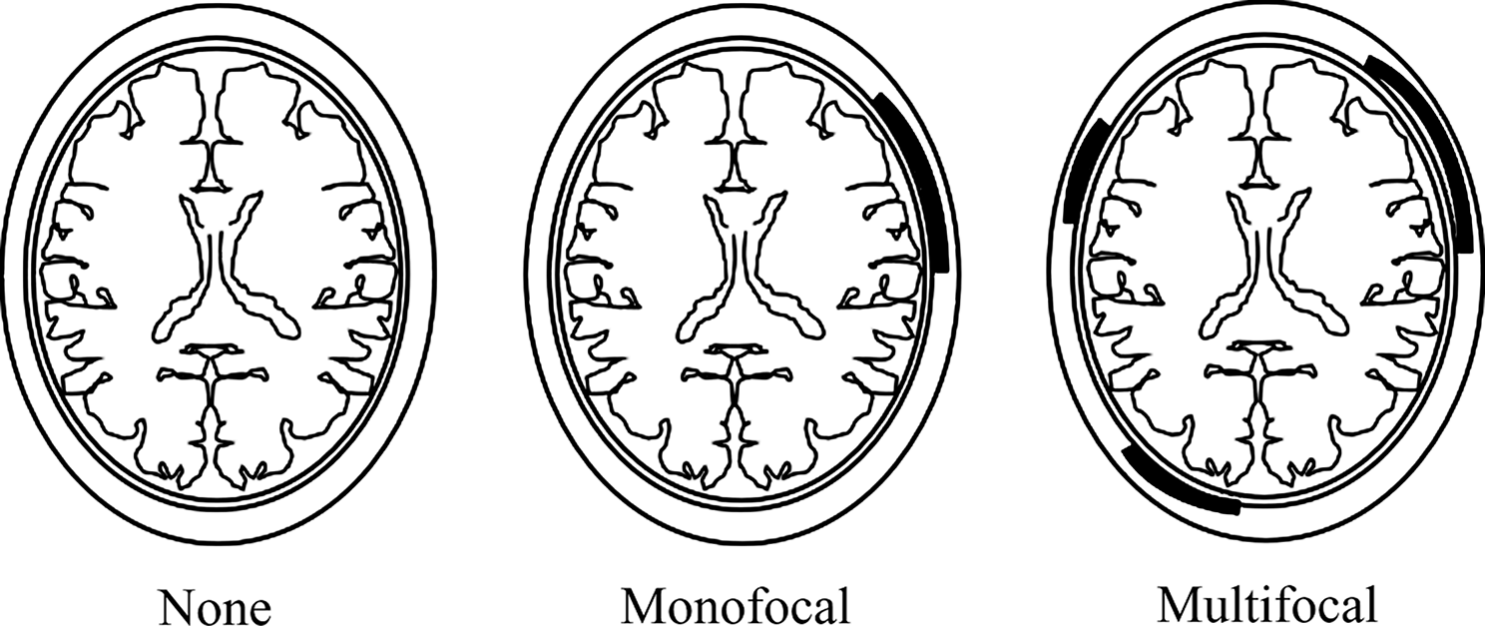
Extracranial abnormal high signal intensity areas on STIR images and abnormal enhancement on contrast-enhanced images were classified as follows: “None,” when no lesions are detected; “Monofocal,” when lesions are detected only in one place; and “Multifocal,” when lesions are detected in multiple places.

Acquired images were retrospectively evaluated by three radiologists (readers A, B, and C, with 14, 10, and 7 years of experience, respectively) simultaneously and independently on PACS. Readers A and B were board-certified radiologists. The readers were blinded to all clinical data. Extracranial abnormal high-signal-intensity areas on STIR images and abnormal enhanced areas on contrast-enhanced images were classified as follows: “None,” when no lesions were detected; “Monofocal,” when lesions were detected only in one place; and “Multifocal,” when lesions were detected in multiple places (Fig. 1). In each sequence, the readers judged the presence of extracranial lesions that were visible in the upper edge of the lateral ventricle up to the infraorbital margin, because subcutaneous fat signal suppression appears to be weak on the top of head, and round or arcuate areas of uniform thickness are rarely observed in the parietal region due to the characteristics of CE-T1W images.

### Statistical analysis

“Non” and “Monofocal” were defined as being negative for the multifocal arcuate sign, whereas “Multifocal” was defined as being positive for the multifocal arcuate sign. Patients were considered to have GCA is the reader made an assessment of “Multifocal.” The sensitivity and specificity of the “multifocal arcuate sign” were calculated for each reader using the cross-tabulation function in Microsoft Excel. Fisher’s exact test was used to compare “Multifocal” findings between five patients with GCA and those with other diseases. *P* < 0.05 was considered statistically significant. In addition, mean Cohen’s kappa and Fleiss’ kappa statistics were used to compare inter-reader agreement, with kappa values being categorized as poor (< 0), slight (0-0.20), fair (0.21–0.40), moderate (0.41–0.60), substantial (0.61–0.80), and almost perfect (0.81-1.00) [12]. All statistical analysis were performed using SPSS ver.26.

## Results

All results are summarized in Table 2. Among the five patients with GCA, two (Cases 10 and 16) were judged as “Multifocal” by all readers, one (Case 8) was judged as “Multifocal” by readers B and C and as “Monofocal” by reader A, and one (Case 14) was judged as “Multifocal” by reader A and as “None” by readers B and C, on both STIR and CE-T1W images. One patient (case 11) was judged as “None” by all readers. Among patients with other diseases, only one (Case 6: postoperative wound infection) was judged as “Multifocal” by reader A on STIR images, while this patient was also judged as “Monofocal” by readers B and C on STIR and CE-T1W images. Five patients with other diseases (Cases 1, 3, 9, 13, and 15) were judged as “Monofocal” by all or some readers, and all other patients (Cases 2, 4, 5, 7, 12, and 17) were judged as “None” by all readers.

**Table 2:** Results of image interpretation by three readers

Case	Diagnosis	Reader A		Reader B		Reader C	
		STIR	CE-T1W	STIR	CE-T1W	STIR	CE-T1W
1	Bone metastasis (gastric cancer)	monofocal	monofocal	monofocal	monofocal	monofocal	monofocal
2	Epilepsy	none	none	none	none	none	none
3	Postoperative temporal hemangioma	none	none	monofocal	monofocal	monofocal	monofocal
4	Aortitis syndrome with headache (not diagnosed as GCA)	none	none	none	none	none	none
5	Epilepsy	none	none	none	none	none	none
6	Postoperative wound infection	**multifocal**	monofocal	monofocal	monofocal	monofocal	monofocal
7	Epilepsy	none	none	none	none	none	none
8	GCA with TAB	monofocal	monofocal	**multifocal**	**multifocal**	**multifocal**	**multifocal**
9	Postoperative wound infection	monofocal	none	none	none	monofocal	monofocal
10	GCA without TAB	**multifocal**	**multifocal**	**multifocal**	**multifocal**	**multifocal**	**multifocal**
11	GCA without TAB	none	none	none	none	none	none
12	Leukemia	none	none	none	none	none	none
13	Extracranial hemangioma	none	none	monofocal	monofocal	monofocal	monofocal
14	GCA without TAB	**multifocal**	**multifocal**	none	none	none	none
15	Leukemia	monofocal	monofocal	none	none	none	monofocal
16	GCA without TAB	**multifocal**	**multifocal**	**multifocal**	**multifocal**	**multifocal**	**multifocal**
17	Leukemia	none	none	none	none	none	none

GCA = giant cell arteritis, TAB = temporal artery biopsy, STIR = short tau inversion recovery, CE-T1W = contrast-enhanced T1-weighted. Extracranial abnormal high-signal-intensity areas on STIR images and abnormal enhanced areas on contrast-enhanced images were classified as follows: “None,” when no lesions were detected; “Monofocal,” when lesions were detected only in one place; and “Multifocal,” when lesions were detected in multiple places

**Fig. 2:**
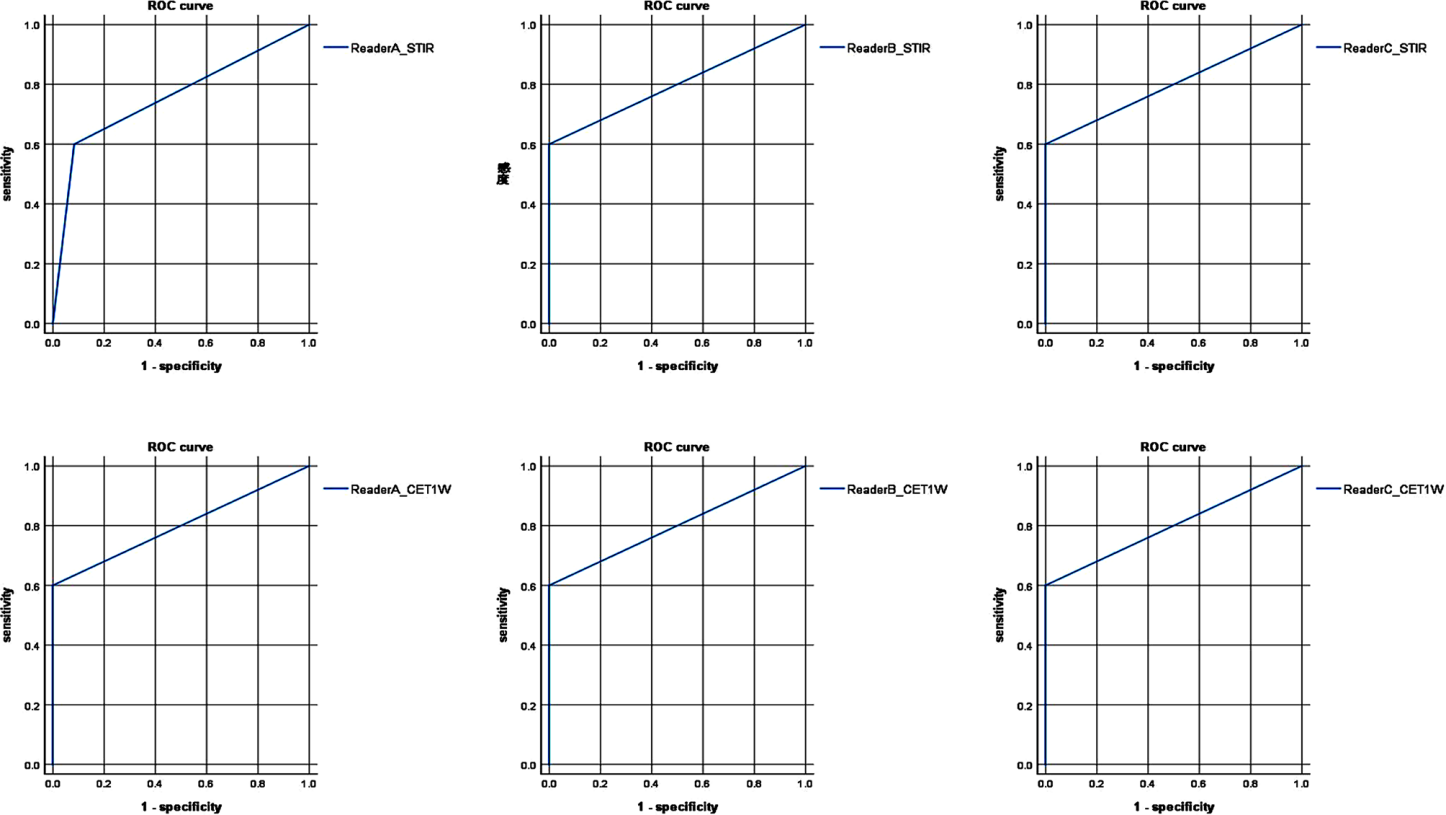
Receiver Operating Characteristic (ROC) curves constructed from results of image interpretation by the three readers in Table 3. Area under the curve (AUC) for ROC curve of reader A on STIR images is 0.758, and other AUCs are all 0.800

Results of image interpretation by the three readers are summarized in Table 3. For reader A, the sensitivity and specificity of the multifocal arcuate sign on both STIR and CE-T1W images in patients with GCA were 60% and 92%, respectively, with positive and negative predictive values of 75% and 85%, respectively. For readers B and C, the sensitivity and specificity of the multifocal arcuate sign on both STIR and CE-T1W images in patients with GCA were 60% and 100%, respectively, with positive and negative predictive values of 100% and 86%, respectively. Fisher’s exact test revealed that significantly more patients with GCA were judged as “Multifocal” compared to those with other diseases (*p* = 0.008–0.027). Receiver Operating Characteristic (ROC) curves were constructed from results of image interpretation by the three readers in Table 3. The area under the curve (AUC) for reader A on STIR images was 0.758, and whereas all other AUCs were 0.800 (Fig. 2). Mean Cohen’s kappa for inter-reader agreement with respect to all 17 patients was 0.54 for STIR sequences and 0.60 for CE-T1W sequences. Mean Cohen’s kappa for inter-reader agreement with respect to five patients with GCA was 0.52 for both STIR and CE-T1W sequences. Fleiss’ kappa for inter-reader agreement with respect to all 17 patients was 0.54 for STIR sequences and 0.60 for CE-T1W sequences, with the kappa value for “Monofocal” on CE-T1W sequences indicating substantial agreement (k = 0.73). Fleiss’ kappa for inter-reader agreement with respect to five patients with GCA was 0.49 for both STIR and CE-T1W sequences, with the kappa value for “Multifocal” indicating moderate agreement (k = 0.44) (Table 4).

**Table 3:** Sensitivity, specificity, positive predictive value, and negative predictive value of the “multifocal arcuate sign” and fisher’s exact test results for each readers

Reader		Sensitivity	Specificity	Positive predictive value	Negative predictive value	Fisher’s exact test
Reader A	STIR	0.6	0.92	0.75	0.85	*p* = 0.027
	CE-T1W	0.6	1	1	0.86	*p* = 0.008
Reader B	STIR	0.6	1	1	0.86	*p* = 0.008
	CE-T1W	0.6	1	1	0.86	*p* = 0.008
Reader C	STIR	0.6	1	1	0.86	*p* = 0.008
	CE-T1W	0.6	1	1	0.86	*p* = 0.008

*Note* STIR = short tau inversion recovery, CE-T1W = contrast-enhanced T1-weighted. “Multifocal” in five GCA patients and “Multifocal” in the other disease were compared using Fisher’s exact test. *P* < 0.05 was considered statistically significant

**Table 4:** Mean Cohen’s kappa and fleiss’ kappa

	All 17 cases		Five GCA cases	
	STIR	CE-T1W	STIR	CE-T1W
Mean Cohen’s kappa	0.54	0.60	0.52	0.52
Fleiss’s kappa				
Overall	0.54	0.60	0.49	0.49
“None”	0.60	0.60	0.70	0.70
“Monofocal”	0.38	0.51	-0.07	-0.07
“Multifocal”	0.63	0.73	0.44	0.44

*Note* STIR = short tau inversion recovery, CE-T1W = contrast-enhanced T1-weighted. Mean Cohen’s kappa and Fleiss’ kappa statistics were used to compare inter-reader agreement, with kappa values being categorized as poor (< 0), slight (0-0.20), fair (0.21–0.40), moderate (0.41–0.60), substantial (0.61–0.80), and almost perfect (0.81-1.00)

### Case presentation of GCA

#### Case 8

**Fig. 3:**
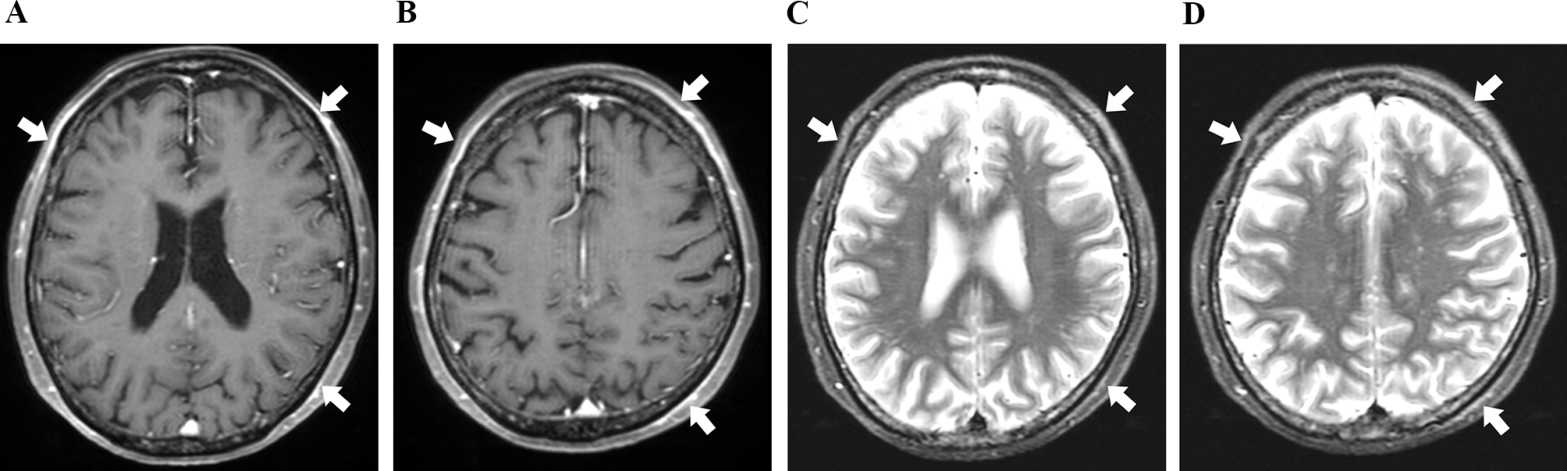
3 Case 8 (giant cell arteritis). **A** and **B**, Contrast-enhanced T1-weighted (CE-T1W) images show multiple distant arcuate enhancement areas of extracranial soft tissues. **C** and **D**, STIR images show multiple distant high signal intensity areas of extracranial soft tissues

A patient with complaints of fever, joint pain, right-side headache, and arthralgia visited. Head MRI was performed, which revealed areas of high signal intensity and contrast enhancement around the right superficial temporal artery. She was diagnosed with GCA on the basis of clinical symptoms and pathological inflammatory findings of temporal artery biopsy. Prednisolone (PSL) was administered, and her symptoms and inflammatory response improved. In addition to high-signal-intensity areas around the biopsied right superficial temporal artery, MRI also revealed an arcuate lesion with enhancement and STIR high signal intensity in multiple locations outside the skull (Fig. 3A and D).

#### Case 10

**Fig. 4:**
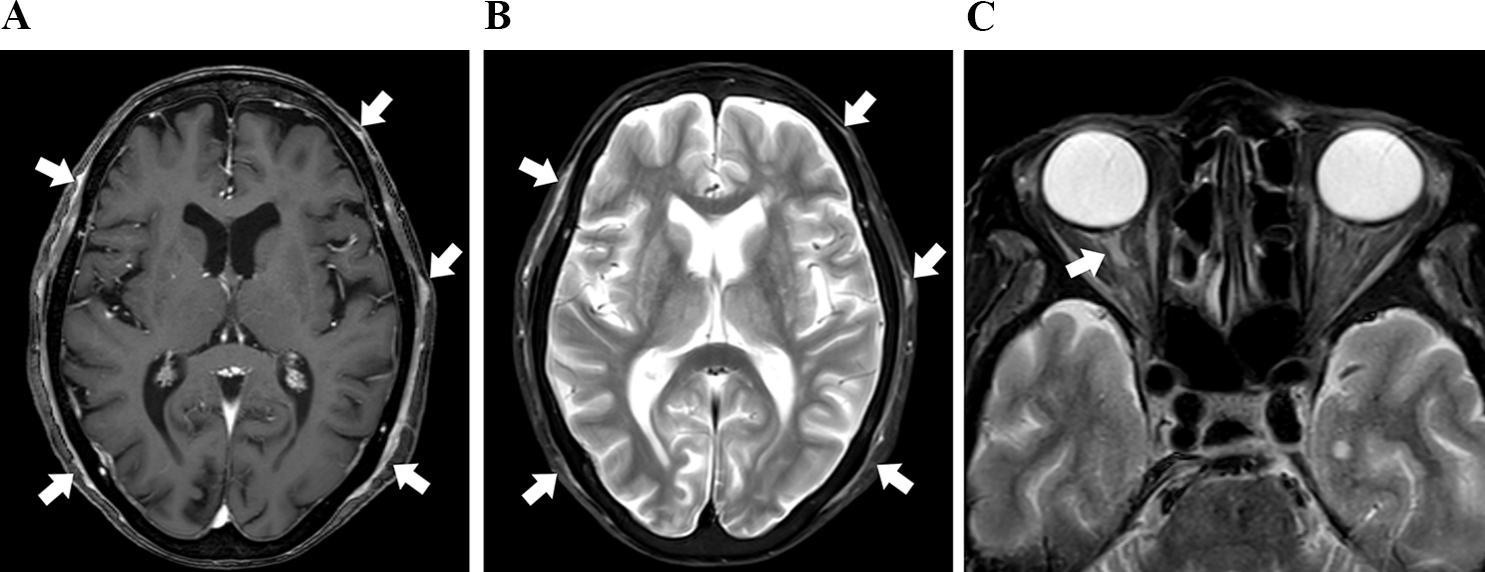
Case 10 (giant cell arteritis). (**a**), Contrast-enhanced T1-weighted (CE-T1W) image shows multiple distant arcuate enhancement areas of extracranial soft tissues. (**b**), STIR image shows multiple distant high signal intensity areas of extracranial soft tissues. (**c**), STIR shows high signal intensity of the right retrobulbar optic nerve

A patient with complaints of headache and deterioration of visual impairment visited another hospital for headache. When she visited our hospital, she had a headache lasting a month, right loss of vision, left decreased vision, and a general sense of coldness. She was suspected to have GCA due to her complaint of unilateral headache. CE-T1W MRI revealed multiple contrast areas around multiple external carotid artery branches (e.g., superficial temporal artery), and STIR high signal intensity was observed in the right retrobulbar optic nerve (Fig. 4C). Based on the clinical and MRI findings, GCA was suspected, and PSL was started. However, her bilateral visual acuity did not improve. Head CE-T1W MRI revealed arched areas with STIR high signal intensity and contrast areas in multiple locations outside the skull (Fig. 4A and B).

#### Case 11

**Fig. 5:**
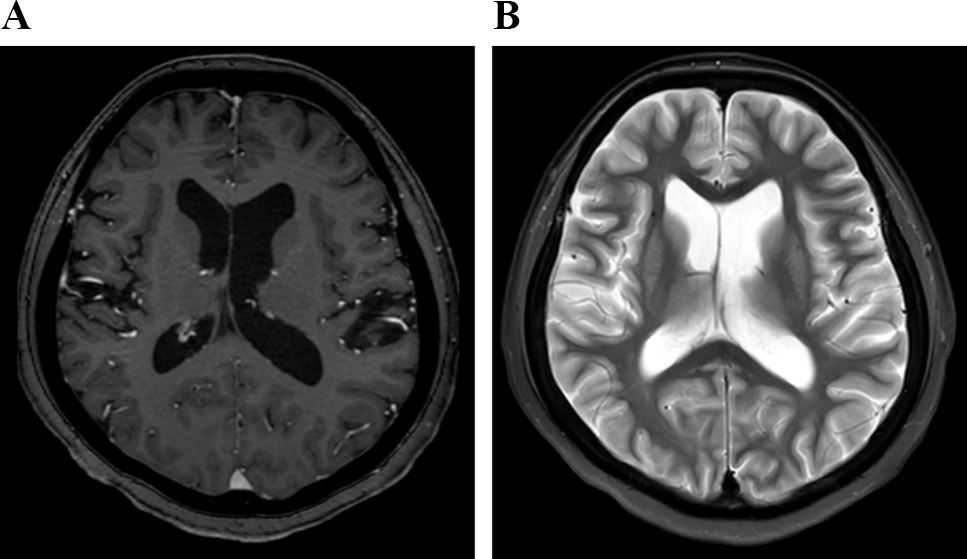
Case 11 (giant cell arteritis). (**a**), Contrast-enhanced T1-weighted (CE-T1W) revealed no abnormal signal area. (**b**), STIR revealed no abnormal signal area

A patient had chief complaints of chills, fever, and headache on the right side. Four months ago, she underwent surgery and outpatient chemotherapy for stage IB endometrial cancer. Contrast-enhanced CT revealed inflammation of the thoracic aorta and tenderness and pulse reduction of the superficial temporal artery; CT, ultrasonography, and blood examinations suggested GCA. PSL was initiated, and her symptoms and inflammatory response improved. Head CE-T1W MRI revealed no abnormal signal area or contrast area on STIR images outside the skull (Fig. 5A and B).

#### Case 14

**Fig. 6:**
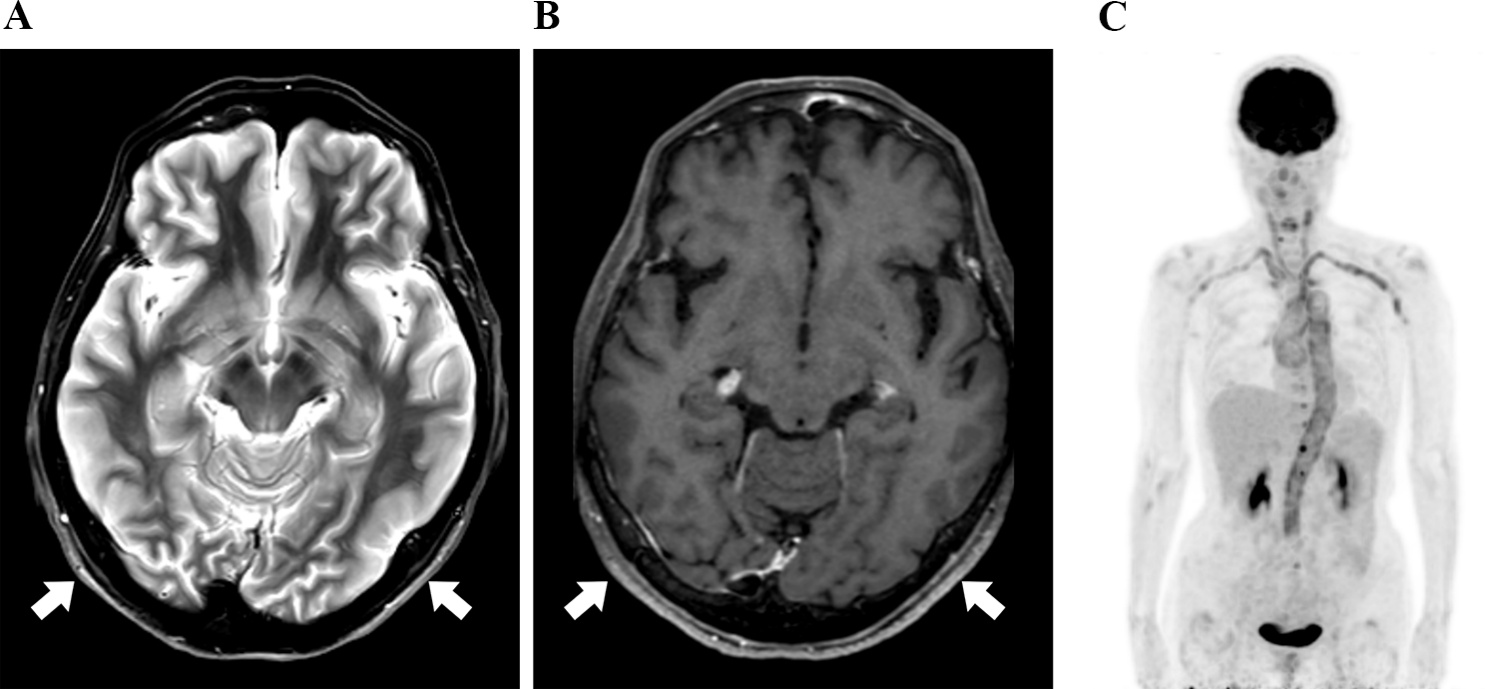
Case 14 (giant cell arteritis). (**a**), STIR image shows high signal intensity areas of posterior extracranial soft tissues. (**b**), Contrast-enhanced T1-weighted (CE-T1W) image shows weak enhancement areas of posterior extracranial soft tissues. (**c**), FDG-PET shows the FDG uptake of aortic wall and subclavian and common carotid arterial wall.

A patient with complaints of fever, nausea, headache, and poor appetite, and with a history of borderline ovarian cancer surgery five years ago with no recurrence visited. She presented with fever and general malaise, which was accompanied by headache and poor appetite. Blood sampling revealed an inflammatory response with high CRP. Contrast-enhanced CT and FDG-PET examinations were conducted to detect the fever focus, and inflammation of the thoracic aorta was observed (Fig. 6C). Combined with the clinical findings, this led to a diagnosis of GCA. PSL was administered, and her symptoms and inflammatory response improved. Wall thickening of the superficial temporal artery had not been pointed out at the time of CE-T1W MRI, but retrospective image examination revealed an arcuate lesion with enhancement and STIR high signal intensity in multiple locations outside the skull (Fig. 6A and B).

#### Case 16

**Fig. 7:**
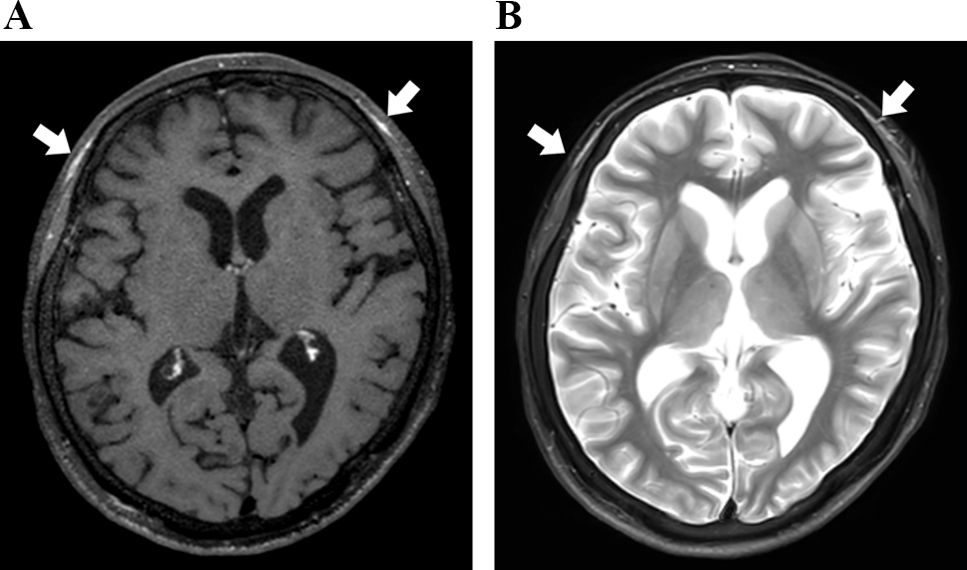
Case 16 (giant cell arteritis). (**a**), Contrast-enhanced T1-weighted (CE-T1W) image shows multiple distant arcuate enhancement areas of extracranial soft tissues. (**b**), STIR image shows high signal intensity areas of anterior extracranial soft tissues

A patient with a complaint of left visual impairment, which appeared five days ago, visited another hospital. When she visited our hospital, it turned out that she had recently noticed a headache. Blood sampling revealed an inflammatory response with high CRP and erythrocyte sedimentation rate, and ultrasonography revealed superficial temporal artery swelling, wall thickening, and tenderness. She was suspected to have GCA. CE-T1W MRI revealed wall thicknessing of the left superficial temporal artery and occipital artery, contrast areas around the left frontal branch of the superficial temporal artery, and arcuate areas with STIR high signal intensity around the bilateral frontal branches of the superficial temporal artery (Fig. 7A and B). She was diagnosed with clinical GCA and treated with PSL. Her inflammation improved, and vision loss did not progress.

## Discussion

This study revealed that the extracranial multifocal arcuate sign, which can be detected on both plain and CE-T1W images, may be used as a new MRI finding for the diagnosis of GCA. We reviewed images of extracranial soft tissues (subcutaneous fat, muscles, and tendons) in patients with GCA to examine the presence of multiple distant arcuate areas with high signal intensity on STIR images and enhancement on CE-T1W images. A previous study reported that typical MRI signs of vascular inflammation in GCA include arterial wall thickening with mural and periadventitial contrast enhancement [9]. However, no study has ever reported on the association between extracranial soft tissue findings and GCA.

GCA and PMR are frequently overlapping diseases, with roughly 50% of patients with GCA presenting with PMR before, at the time of, or after the diagnosis of GCA [3, 4]. PMR is characterized by pain and stiffness involving the shoulder girdle, proximal aspects of the arms, the neck, and the pelvic girdle [4]. MRI of shoulder joints reportedly show increased thickening of the supraspinatus tendon in patients with PMR, which is likely to coexist with GCA, compared to rheumatoid arthritis [13]. This may be because a combination of PMR and vasculitis is associated with stronger inflammatory findings than those of rheumatoid arthritis. Strong inflammation of extracranial soft tissues due to PMR and inflammation of the vascular wall of arteries possibly result in multifocal enhancement and STIR high signal intensity in extracranial soft tissues. This extracranial finding, referred to as the multifocal arcuate sign, may be regarded as a new radiologic finding of GCA on STIR and post-contrast images.

In a previous study, contrast-enhanced MRI of superficial cranial arteries in the initial diagnosis of GCA had a diagnostic accuracy of 78.4% in terms of sensitivity and 90.4% in terms of specificity, on the basis of mural wall thickening and the signal intensity of mural-periadventitial contrast enhancement in six arterial segments (the frontal and parietal branches of the superficial temporal artery and the occipital artery bilaterally) [9]. The sensitivity of our diagnostic method was lower compared to existing methods in contrast-enhanced sequences, and the sensitivity, specificity, and inter-reader agreement of the multifocal arcuate sign for diagnosing GCA were low. However, considering that GCA is not currently diagnosed by plain MRI, the multifocal arcuate sign is a potentially powerful radiographic finding that may make plain MRI a promising modality with high diagnostic accuracy. Importantly, the multifocal arcuate sign can be confirmed on STIR images (i.e., without contrast). Radiographic findings of GCA in previous studies were obtained from contrast-enhanced MRI [8, 9]. Our findings suggest that the new finding may contribute to the early diagnosis of GCA, i.e., at the screening stage by plain MRI. GCA is an important disease, and its early diagnosis and early introduction of steroid treatment can prevent blindness, aortic dissection, aneurysm formation, myocardial infarction, and cerebrovascular accidents. The multifocal arcuate sign may help solve the problem of diagnostic delay in GCA.

This study has several limitations. First, the study was performed in a single institute with a small sample size. GCA is a relatively rare disease, and few patients undergo both contrast-enhanced MRI and whole-brain axial STIR imaging simultaneously. Although the number of patients in this study may not have been sufficient for a robust statistical analysis, we believe it important to present the sensitivity, specificity, PPV, and NPV of the multifocal arcuate sign for future use and validation. Therefore, we performed a statistical analysis based on the limited number of cases in this study. Further investigation is warranted to verify our findings in a larger cohort of patients with GCA and those with other diseases, for whom both contrast-enhanced and STIR images are available. Second, we used two sequences with suppressed fat signal for contrast-enhanced MRI, i.e., T1-TFE and T1-VISTA sequences. These sequences have different properties regarding the effect of intravascular signal suppression, and it is desirable to use T1-VISTA rather than T1-TFE to determine vessel wall thickness and enhancement of the arterial vessel wall. However, it is unlikely that the existence of the intravascular signal suppression effect was problematic in the evaluation of extracranial soft tissues, such as subcutaneous fat, muscles, and tendons, which can be evaluated without the intravascular signal suppression effect. Third, GCA was clinically diagnosed without biopsy in all but one patient.

When GCA is suspected because the patient has temporal pain, fever, headache, arthralgia, or rapidly progressing vision loss, the presence of the multifocal arcuate sign using STIR and CE-T1W images will be useful in the early diagnosis of GCA.

## Conclusion

We examined the “multifocal arcuate sign” as a new radiographic finding of GCA on both STIR and contrast-enhanced sequences. Our findings suggest that this “multifocal arcuate sign” may be a powerful radiographic finding that can make plain MRI a promising diagnostic modality.

## Data Availability

All data relevant to the study are included in the article.
